# Occult Congenital Ureteropelvic Junction Obstruction in Two Adults Presenting with Collecting System Rupture After Blunt Renal Trauma: A Case Report Series

**DOI:** 10.1089/cren.2015.0016

**Published:** 2015-12-01

**Authors:** Haley E. Hoffner, Lawrence M. Dagrosa, Eric P. Raffin, Vernon M. Pais

**Affiliations:** ^1^ College of Medicine, State University of New York Downstate Medical Center, Brooklyn, New York.; ^2^ Department of Urology, Dartmouth-Hitchcock Medical Center, Lebanon, New Hampshire.

## Abstract

We report two adult cases of congenital ureteropelvic junction obstruction detected incidentally in the setting of blunt abdominal trauma. CT images are provided to describe the presentation, while review of the literature and management of renal trauma are discussed.

## Introduction

Congenital ureteropelvic junction (UPJ) obstruction occurs in 1 in 2000 children.^1^ The physiologic sequela of chronic obstruction is well understood with increased pressure within the collecting system transmitting to the renal tubules and glomerulus, ultimately leading to decreased renal blood flow, tubulointerstitial fibrosis, and obstructive uropathy.^2–4^ Less commonly discussed is the increased risk for traumatic injury in these anomalous hydronephrotic kidneys. It has been reported that up to 19% of injuries occurring from blunt renal trauma occur in pathologic kidneys.^5^ The majority of the published literature on the topic, however, has focused primarily on the pediatric population.^6^ In this study, we present two unusual cases of adult blunt renal trauma, resulting in collecting system rupture in the setting of severe hydronephrosis with presumed, underlying, and undiagnosed congenital UPJ obstruction.

## Case Report 1

A healthy 25-year-old gentleman with no significant medical history presented to the emergency department with a 6-hour history of abdominal pain that started immediately after being punched in the flank during a hockey fight. He was able to skate to the bench but unable to continue playing due to the pain. He then developed severe nausea and vomiting while driving home, which prompted his presentation to the emergency department. Questioning revealed an uncomplicated birth history with standard prenatal care. He denied any history of urinary tract infections and abdominal or flank pain before this presentation.

Physical examination revealed a hemodynamically stable well appearing gentleman in no apparent distress. His abdomen was soft, nontender, and nondistended without organomegaly or abdominal herniation. External genitalia examination revealed a normal phallus with no rash or skin changes, no blood at the meatus, as well as no scrotal swelling or erythema. Furthermore, the patient was able to void without problems while in the emergency department.

Laboratory results were remarkable for an elevated creatinine of 1.9. There was no microscopic hematuria on urinalysis.

**FIG. 1.**
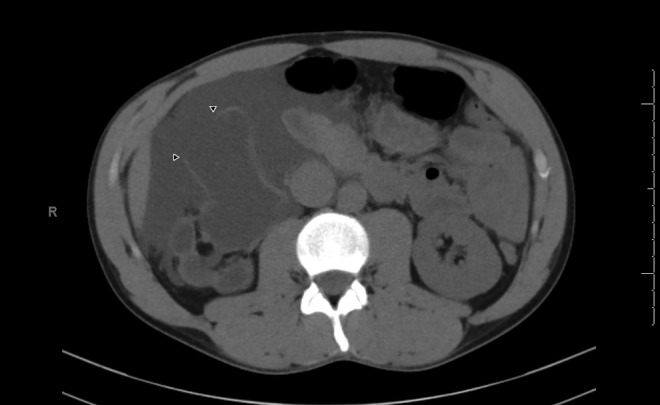
CT abdomen without contrast; axial image of right kidney demonstrating rupture (*arrow heads*) of a severely hydronephrotic pelvis with associated severe cortical atrophy and urine extravasation.

Abdominal CT without contrast demonstrated a large, low-density retroperitoneal fluid collection consistent with urine that extended along the right paracolic gutter and into the pelvis. Also noted was a defect in the right renal pelvis, which was severely dilated and associated with severe right cortical atrophy (Fig. 1). These findings were consistent with a Grade IV renal trauma according to the Revised Renal Injury Staging Classification.^7^ There were no other traumatic findings of the abdomen and pelvis.

Urology was consulted and recommended 24 hours of observation to ensure that the patient remained hemodynamically stable and pain remained well controlled. His severe cortical atrophy was interpreted as undiagnosed congenital UPJ obstruction with long-standing obstruction and his elevation in creatinine as reabsorption of extravasated urine. Acute intervention was deemed unnecessary, given the inferred functional status of his right kidney; significant bleeding and expansion of the urinoma were unlikely. Percutaneous decompression and need for future characterization of his UPJ were unlikely to be of use, given the presumed minimal contribution of his injured kidney to his overall renal function. His hospital stay was without event, and he was discharged the following day with outpatient follow-up with urology in 6 weeks for symptom check and abdominal ultrasound.

## Case Report 2

A healthy 44-year-old woman with no significant medical history presented to the emergency department with a 4-hour history of abdominal pain following a mountain biking accident. She had been biking downhill when she pumped the brakes too hard, causing her to fall forward off her bike and hit her left abdomen on the handle bars. Although the fall was routine for outdoor activity and seemingly minor, she immediately developed severe lower quadrant abdominal pain, which prompted her to seek the nearest emergency department. Upon questioning, she reported an uncomplicated birth history as well as regular pediatric care. She denied history of gross hematuria, urinary tract infections, and abdominal or flank pain before this admission.

Physical examination revealed vital signs within the normal range and a well appearing woman in no apparent distress. Her abdomen was tender in the left upper quadrant, but soft, nondistended, and without rash or ecchymosis. Her flanks were nontender and without ecchymosis. Her skin was intact with no evidence of trauma, including laceration, abrasion, or contusion. Her pelvis was stable to anteroposterior and lateral compression. External genital examination revealed normal urethral meatus and vaginal introitus without erythema, edema, or blood. A foley was placed and returned clear light yellow urine.

Laboratory studies were remarkable only for leukocytosis of 16,000. There was no microscopic hematuria on urinalysis.

**FIG. 2.**
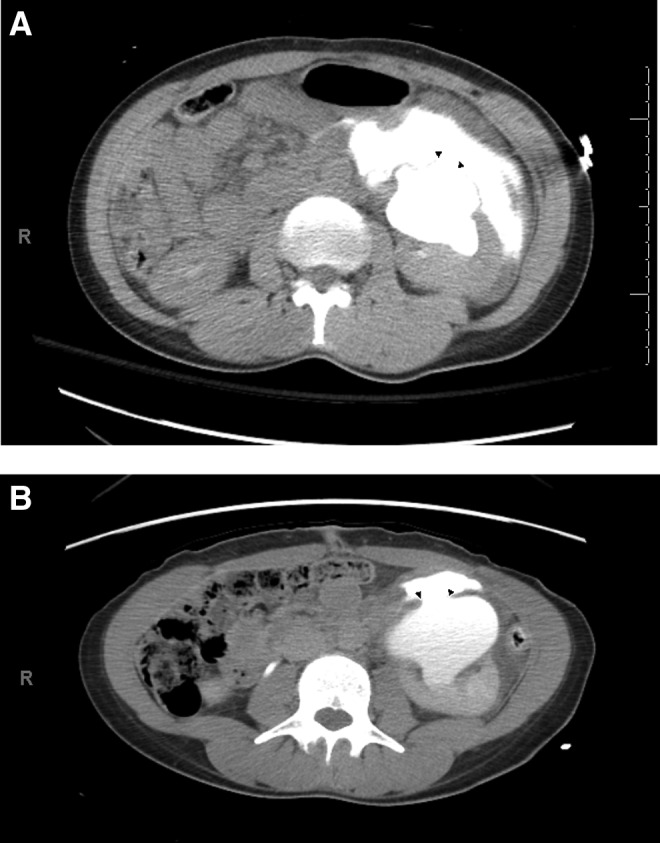
**(A)** Delayed phase of CT urogram; axial image of left kidney showing contrast extravasation and 11 mm defect (*arrow heads*) in the renal pelvis. **(B)** Delayed phase of CT urogram; axial image of left kidney showing persistence of pelvic defect (*arrow heads*) with contrast extravasation, but marked interval decrease in urinoma after percutaneous nephrostomy tube placement.

Abdominal CT with intravenous contrast revealed pronounced dilation of the left renal collecting system with a large collection of retroperitoneal fluid. The delayed phase showed contrast opacification of the fluid collection and a ∼13 mm defect in the anterior left renal pelvis (Fig. 2A). No contrast was appreciated within the left ureter, preventing its assessment. These findings were consistent with a renal pelvis rupture with urinary extravasation, a Grade IV renal trauma according to the Revised Renal Injury Staging Classification.^7^ There were no other traumatic findings.

Urology was consulted. Her severe hydronephrosis with the absence of any contrast excretion into the nondilated ureter on delayed imaging was interpreted as undiagnosed congenital UPJ obstruction with long-standing obstruction. In contrast to the above case, the renal parenchyma associated with the severe hydronephrosis was appearing normal. Percutaneous nephrostomy drainage was recommended as it would allow for decompression of the collecting system, diversion of urine away from the ruptured segment of renal pelvis, and improved likelihood of healing without surgical intervention. Furthermore, a nephrostomy tube would allow for better characterization of the presumed UPJ obstruction in the future. Interventional radiology placed a left percutaneous nephrostomy tube under ultrasound guidance without complication.

During inpatient care, she remained afebrile and hemodynamically stable. On her third hospital day, a repeat CT urogram was obtained to reassess her injury and revealed persistence of the defect in the left anterior renal pelvis, but with marked interval decrease in the urinoma (Fig. 2B). She was discharged home with outpatient follow-up with urology to further evaluate her presumed UPJ obstruction and discuss further management.

## Comment

With the widespread use of prenatal screening ultrasonography, hydronephrosis is typically detected *in utero*. As one of the most frequent fetal abnormalities, the significance of such prenatal hydronephrosis has been a topic of much research,^8^ with 25% of cases resolving spontaneously and a small percentage of persistent cases requiring surgical intervention.^9^ Consequently, hydronephrosis detected *in utero* is typically followed to ensure proper management and preservation of kidney function.^10^ The present cases are striking in the occult nature of the UPJ obstruction and its presentation. Similar cases reported in the literature of blunt trauma uncovering chronic obstruction and hydronephrosis have thus far included only pediatric patients.^11,12^

The management of renal trauma has changed dramatically over the past few decades from operative exploration to nonoperative management.^13^ This trend applies to cases of high-grade blunt injury,^14,15^ including isolated urinary extravasation, and ureteropelvic disruption with urinary extravasation has been effectively managed with an exclusively nonoperative approach.^16^ The collection of ruptured sterile urine poses no immediate threat and is resorbed over time; endoscopic instrumentation may contaminate the urinoma^17^ and should be reserved for either symptomatic patients or for patients who develop complications.

Our first patient had severe cortical atrophy with a correspondingly atrophic blood supply, making the suspicion for urine production with persistent extravasation or retroperitoneal bleeding low. He received observation and supportive care exclusively, without development of symptoms or complication as an inpatient or at outpatient follow-up. Our second patient in contrast had healthy appearing renal parenchyma on the affected side, suggesting high-flow urine production. Urine flow through the path of least resistance would likely compromise healing of the defect. Consequently, our second patient received percutaneous nephrostomy to divert urine away from the injured segment of renal pelvis. Nephrostomy was the preferred choice over stenting in her particular case so that instrumentation would not obscure her presumed UPJ obstruction and to allow further characterization in the future.

We reported two cases in which long-standing asymptomatic hydronephrosis secondary to congenital UPJ obstruction was uncovered by renal pelvis rupture after minor trauma. These cases were effectively treated with conservative intent and serve as support for the trend toward noninvasive management, even in an unorthodox situation.

## Abbreviations Used

CT: computed tomography
UPJ: ureteropelvic junction
